# Resting heart rate variability and exercise capacity in Type 1 diabetes

**DOI:** 10.14814/phy2.13248

**Published:** 2017-04-18

**Authors:** Luke C. Wilson, Karen C. Peebles, Neil A. Hoye, Patrick Manning, Catherine Sheat, Michael J. A. Williams, Gerard T. Wilkins, Genevieve A. Wilson, James C. Baldi

**Affiliations:** ^1^ Department of MedicineUniversity of OtagoDunedinNew Zealand; ^2^ HeartOtagoUniversity of OtagoDunedinNew Zealand; ^3^ Cardiovascular Systems LaboratoryUniversity of OtagoWellingtonNew Zealand; ^4^ Department of PhysiologyUniversity of OtagoDunedinNew Zealand; ^5^ Department of Health ProfessionsFaculty of Medicine and Health SciencesMacquarie UniversityNew South WalesAustralia

**Keywords:** Autonomic nervous system, HR reserve, type 1 Diabetes, *V̇*O_2max_

## Abstract

People with type 1 diabetes (T1D) have lower exercise capacity (*V̇*O_2max_) than their age‐matched nondiabetic counterparts (CON), which might be related to cardiac autonomic dysfunction. We examined whether Heart Rate Variability (HRV; indicator of cardiac autonomic modulation) was associated with exercise capacity in those with and without T1D. Twenty‐three participants with uncomplicated T1D and 17 matched CON were recruited. Heart rate (HR; ECG), blood pressure (BP; finger photo‐plethysmography), and respiratory rate (respiratory belt) were measured during baseline, paced‐breathing and clinical autonomic reflex tests (CARTs); deep breathing, lying‐to‐stand, and Valsalva maneuver. Baseline and paced‐breathing ECG were analyzed for HRV (frequency‐domain). Exercise capacity was determined during an incremental cycle ergometer test while *V̇*O_2_, 12‐lead ECG, and BP were measured. In uncomplicated T1D, resting HR was elevated and resting HRV metrics were reduced, indicative of altered cardiac parasympathetic modulation; this was generally undetected by the CARTs. However, BP and plasma catecholamines were not different between groups. In T1D, *V̇*O_2max_ tended to be lower (*P* = 0.07) and HR reserve was lower (*P* < 0.01). Resting Total Power (TP) had stronger positive associations with *V̇*O_2max_ (*R* ^2^ ≥ 0.3) than all other traditional indicators such as age, resting HR, and self‐reported exercise (*R* ^2 ^= 0.042–0.3) in both T1D and CON. Alterations in cardiac autonomic modulation are an early manifestation of uncomplicated T1D. Total Power was associated with reduced exercise capacity regardless of group, and these associations were generally stronger than traditional indicators.

## Introduction

People with type 1 diabetes (T1D) have poorer exercise capacity than their aged‐matched nondiabetic counterparts (Hilsted et al. 1979; Kahn et al. 1986; Roy et al. 1989; Hagglund et al. 2012; Peltonen et al. 2012; Koponen et al. 2013; Rissanen et al. 2015). In nondiabetics autonomic function is associated with exercise capacity (see review (Hautala et al. 2009)). It is common for T1D to suffer from cardiac autonomic dysfunction and/or neuropathy (i.e., changes in sympathetic (SNS) or parasympathetic (PNS) modulation or both (Vinik and Ziegler 2007; Pop‐Busui 2010; Karayannis et al. 2012; Vinik et al. 2013)), and this may contribute to poorer exercise capacity. Common manifestations of cardiac autonomic dysfunction and/or neuropathy are an elevation in resting heart rate (HR) and a reduction in maximum (max) HR, hence a lower HR response to exercise (i.e., reduced HR reserve). Previous studies report that exercise capacity is significantly reduced in those with “complicated” T1D (i.e., with concomitant diabetes‐related chronic disease) and known cardiac autonomic neuropathy (Hilsted et al. 1979; Kahn et al. 1986; Roy et al. 1989). However, cardiac autonomic damage is a progressive phenomenon and subclinical manifestations such as an imbalance of cardiac autonomic modulation can occur in the early stages of T1D, with prognostic implications (Vinik and Ziegler 2007; Pop‐Busui 2010; Karayannis et al. 2012; Vinik et al. 2013). To date little is known about the association between cardiac autonomic modulation and exercise capacity in those with uncomplicated T1D (i.e., free from known diabetes‐related chronic disease). Indeed understanding whether there is a relationship between impaired cardiac autonomic modulation and reduced exercise capacity may offer further prognostic utility in the T1D population.

Resting HR and analysis of its variability (HRV, Heart rate variability) are commonly used in the clinical and research areas to gain insight into cardiac autonomic modulation. HRV, measured for a short time period in the frequency‐domain, yields four inter‐related metrics of variability, which are reported as power. Specifically, Total Power (TP), High Frequency Power (HF), and Low Frequency Power (LF), and the LF/HF ratio are quantified (Task Force, 1996). Care needs to be taken when interpreting these metrics, as there is controversy as to what they represent (Eckberg 1997; Parati et al. 2006; Reyes del Paso et al. 2013; Joyner 2016). Nevertheless, advantages of this technique are that it is non‐invasive, requires minimal patient input and in the general sense provides a prognostic insight into global cardiovascular health (Task Force, 1996; Thayer and Lane 2007; Reyes del Paso et al. 2013). Specifically, lower TP, HF and LF are associated with increased cardiovascular morbidity and mortality in the general (Tsuji et al. 1994, Task Force, 1996; Thayer and Lane 2007; Joyner 2016) and T1D populations (Colhoun et al. 2001; Vinik and Ziegler 2007). Indeed HRV analysis has recently been recommended as an adjuvant to the traditional clinical autonomic reflex tests (CARTs), in T1D (Bernardi et al. 2011; Karayannis et al. 2012).

To the best of our knowledge only one study has investigated whether HRV is associated with reduced exercise capacity in patients with uncomplicated T1D. Hagglund et al. (2012) investigated HRV in 10 T1D and found no association between resting HRV metrics and *V̇*O_2max_ in this group. However, the absence of association was assessed with a small sample size (*n* = 10) and with second derivative frequency‐domain metric (i.e., derived from TP); HF power. Accordingly, the relationship between resting HRV and exercise capacity in T1D requires further investigation.

Hence, the aim of this study was to examine resting HRV and whether it was associated with exercise capacity in people with and without uncomplicated T1D. Emphasis was placed on TP as it is a global indicator of HRV (Task Force, 1996, Bernardi et al. 2011). We hypothesized that resting TP would be positively associated with exercise capacity in people with or without uncomplicated T1D. Associations between exercise capacity variables and the LF/HF ratio, a controversial reflection of autonomic balance (Parati et al. 2006) were also considered.

## Methods

The study was approved by the Lower South Health and Disability Ethics Committee, New Zealand, and complied with the *Declaration of Helsinki*. All participants gave written informed consent. In participants with T1D this included access to their clinical records for their latest HbA_1c_ measurement. The latter reflects average glycemia over the preceding 3 months.

### Participants

Participants comprised 23 T1D and matched 17 nondiabetics (CON; see Results for details of matching). The T1D participants were recruited from the Endocrinology Clinic at the Dunedin Public Hospital, and the CON participants were recruited from the local community. Prior to acceptance into the study participants completed a health screening questionnaire [documenting: smoking history, diagnosis of diabetes, self‐reported exercise (i.e., hours of physical activity per week), medication use, and comorbidities]. Exclusion criteria were known diabetic neuropathy (autonomic and peripheral) and nephropathy, as well as cardiovascular, cerebrovascular, and respiratory disease, and medications which affect HR (i.e., beta‐blockers).

### Experimental design

All experiments were conducted within the Dunedin Public Hospital. Participants arrived at the laboratory between 8 am and 2 pm, with each experimental session lasting 2–3 h, ~2 h after eating a non‐standardized light meal, and having abstained from caffeine, alcohol, and heavy exercise for at least 12 h (Bernardi et al. 2011; Spallone et al. 2011). The experimental protocol comprised two successive parts, Part 1 always preceded Part 2, due to the aforementioned standardization requirements. Part 1 (~1 h) involved measurement of baseline and paced breathing parameters and CARTs, generally ordered from those that cause the smallest physiological perturbations to the largest (see below). This was conducted in a research laboratory. Part 2 involved a maximal exercise capacity test. This was conducted in a specialized clinical laboratory, with standard emergency equipment. Participants voided their bladders before commencing the experimental protocol.

#### Part 1: Measurement of resting and paced breathing parameters and CARTs

Participants were instrumented in a supine position as follows. A 3‐lead electrocardiograph [(ECG; Lead II position); FE132, ADInstruments, Dunedin, NZ] was used to measure heart rate (HR) and rhythm. Finger photoplethysmography (Finometer^®^ MIDI, FMS, Finapres Medical Systems BV, the Netherlands) was used to measure beat‐to‐beat arterial blood pressure (BP). The finometer cuff was referenced to heart level and measurements were verified against manual sphygmomanometer (ADC DIAGNOSTIX™ 972, ADC, NY) measurements, taken on the contralateral arm. A respiratory belt transducer (MTL1132, ADInstruments, Dunedin, NZ) was placed around the chest to detect breathing frequency. All data these signals were recorded continuously at 1 kHz via an analog‐to‐digital converter (Powerlab/3508/P; ADInstruments, Dunedin, NZ) and stored for offline analysis.

##### Baseline parameters

After acclimation to the equipment (~15 min supine rest) 5 min of baseline (resting) data was collected. Participants were instructed not to speak and to minimize movement during data collection. Baseline HR, breathing frequency and BP were determined by averaging the final 3 min of the baseline recording. In addition, the ECG data was used in HRV analysis (see below).

##### Paced breathing

Participants performed 5 min of paced breathing guided by a metronome at a rate of 15 br/min (2 sec inspiration, 2 sec expiration). During which participants were instructed not to increase their breathing depth (i.e., avoid hyperventilation). Breathing frequency was controlled at the prescribed rate in both groups (T1D: 14.9 ± 0.5 br/min and CON: 14.9 ± 0.5 br/min). The final 3 min of paced breathing ECG data was also used in HRV analysis.

##### HRV analysis

HRV analysis was performed in accordance with the Task Force of the European Society of Pacing and Electrophysiology (Task Force, 1996). The ECG was screened for artefacts, and frequency‐domain HRV was determined using the Fast‐Fourier Transform method (HRV module v1.4.2, ADInstruments, Dunedin, NZ). The HRV metrics obtained (in msec^2^) were TP (0–0.4 Hz) and its derivatives, Very‐Low Frequency Power (VLF: 0–0.04 Hz), LF (0.04–0.15 Hz), and HF (0.15–0.4 Hz). The LF/HF ratio was then calculated. In this study, the VLF was not presented as it is unreliable in short term recordings (Task Force, 1996). Time‐domain HRV was not determined, as there is a concern with its sensitivity and reproducibility with the shorter collection times used within this study (Task Force, 1996).

##### CARTs

Four standard clinical tests and analysis approaches were used to assess cardiovascular autonomic modulation and a composite criteria (see below) of abnormal values/responses were used to detect the possible presence cardiovascular autonomic dysfunction as detailed in the current consensus statements (see; (Bernardi et al. 2011; Spallone et al. 2011)). These were the assessment of resting HR, the response to deep breathing, lying‐to‐standing, and a Valsalva maneuver which are detailed below. Each of the tests (or repeat tests) were separated by at least 5 min for recovery (Bernardi et al. 2011; Spallone et al. 2011). (1) Resting HR, which was obtained during baseline for each participant, was screened for the presence (abnormal) or absence of tachycardia (defined as HR > 100 b/min). (2) Deep breathing and the magnitude of the respiratory‐evoked HR change. Participants guided by a metronome were instructed to breathe 6 br/min (5 sec inspiration, 5 sec expiration) for 1 min, to a depth that equaled their vital capacities. This occurred twice separated with the recovery period. For analysis, the highest HR (occurs during inspiration) and the lowest HR (occurs during expiration) were determined separately for each breath and across each trial. Then the maximum change in HR was determined by difference between the mean of the three highest and lowest HR's in each trial. The trial which was determined to have the maximal HR response and optimal breathing control was selected, and a HR change of <10 b/min was taken to reflect abnormal cardiac autonomic modulation. (3) Supine‐to‐standing and the posture change induced HR and BP response. After 1 min collection of supine data, participants were instructed to rapidly stand from a supine position, and remain there as still as possible for 3 min, from this two parameters were determined. (a) the 30:15 ratio for the HR response where the longest R‐R interval (20–40th beats) is divided by the shortest R‐R interval (5–25th beats) after standing. For each participant the 30:15 ratio was compared to age‐adjusted normative values to reflect normal or abnormal cardiac autonomic modulation (Spallone et al. 2011); and (b) the systolic BP response to standing, where the systolic BP (average for 1 min) following ≥2 min of standing was subtracted from the mean systolic BP from the preceding supine period. A fall in systolic BP of >30 mm Hg was taken to reflect cardiovascular autonomic modulation. (4) The Valsalva maneuver and the alterations in intrathoracic pressure to evoke changes in HR. Participants in the supine position, were instructed to breathe in as deeply as possible before forcefully exhaling into a closed mouthpiece to generate an expiratory pressure of 40 mm Hg for 15 sec. The time (T1D: 16.7 ± 0.7 sec and CON: 16.7 ± 1.5 sec) of and the generation of expiratory pressure (T1D: 39 ± 8 mm Hg and CON: 39 ± 6 mm Hg) was measured by a calibrated pressure transducer (Model: P‐23 ID, Statham Instruments, Inc). Following the maneuver, the participants were instructed to resume normal breathing. The Valsalva maneuver was repeated at least once, with the maneuver closest to the fore mentioned time and pressure criteria and with the most consistent pressure generation selected for analysis. The Valsalva ratio was determined as the shortest R‐R interval during the maneuver divided by the longest R‐R interval within a minute following the maneuver. The values obtained for each participant were compared to age‐adjusted normative values to reflect normal or abnormal cardiac autonomic modulation (Spallone et al. 2011).

The number of abnormal results denoted possible cardiovascular autonomic dysfunction and/or neuropathy. Specifically: (1) one abnormal HR test suggested early cardiovascular autonomic dysfunction and/or neuropathy; (2) two or three abnormal HR tests confirmed cardiovascular autonomic dysfunction and/or neuropathy; and (3) two or three abnormal HR tests plus an abnormal systolic BP response to standing to reflected severe involvement of cardiovascular autonomic control (Bernardi et al. 2011; Spallone et al. 2011).

#### Part 2: Maximal exercise capacity

Prior to maximal exercise testing participants had a cannula inserted in a antecubital vein, and were then instrumented with a standard diagnostic 12‐lead ECG. Then they rested (supine) for 20 min and before a 10 mL blood sample was taken (following ~3 mL discarded), the sample was taken into chilled EDTA vacutainers and placed on ice (~5 min) before processing (see below). The cannula was then flushed with sterile normal saline (0.9% NaCl) to maintain patency. Participants then performed a maximal incremental exercise test on a cycle ergometer (Model: E100 P, COSMED), while BP, ECG and metabolic variables [*V̇*O_2_, *V̇*CO_2_, and R (i.e., *V̇*CO_2_/*V̇*O_2_); Quark CPET, COSMED)] were monitored and measured at each stage. The protocol started at 50 W and increased at a magnitude of 25 or 50 W (individualized for each participant) every 2 min, until volitional exhaustion. In the 30 sec prior to volitional exhaustion, a 10 mL exercise blood sample (using the procedure detailed above) was acquired from each participant.

A successful exercise test which allowed for determination of *V̇*O_2max_ was indicated by at least two of the following criteria; an R value of ≥1.1; a max HR ≥85% of age‐predicted max (220 – age); a plateau in O_2_ consumption (<150 mL/min) despite an increase in workload; and a failure to maintain the workload (Edvardsen et al. 2014). The *V̇*O_2max_ was determined as the mean the three highest consecutive *V̇*O_2_ values, max HR as the highest obtained HR, and then HR reserve was calculated as max HR – resting HR (obtained in the analysis of part (1). In addition, the maximum breathing frequency, tidal volume, ventilation and R, were recorded. T1D participants had their blood glucose checked prior to and after exercise.

##### Blood analysis

The blood samples (rest and exercise) were centrifuged separately at 1500 G for 15 min at 4°C, the plasma extracted, and frozen and stored in a −80°C freezer for later batch analysis. Plasma noradrenaline and adrenaline was extracted using aluminum oxide technique and analyzed using high‐performance liquid chromatography with electrochemical detection (Eisenhofer et al. 1986).

### Statistical analysis

**Figure 1:**
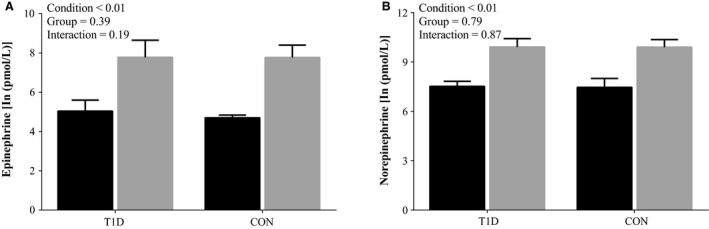
Plasma catecholamines at baseline and maximal exercise. (A) Epinephrine; (B) Norepinephrine. Baseline (black bars) and maximum exercise (gray bars), in controls (CON;* n *=* *13) and people with Type 1 Diabetes (T1D; *n *=* *15). Values are natural log transformed and are presented as means ± SD.

**Figure 2:**
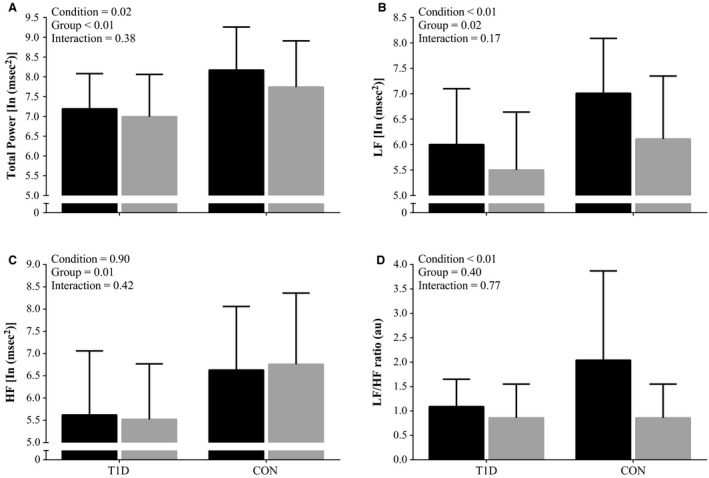
Heart Rate Variability indices during baseline and paced breathing. During baseline (black bars) and paced breathing (gray bars), in controls (CON;* n *=* *17) and people with Type 1 Diabetes (T1D; *n *=* *23). (A) TP; Total Power. (B) LF; Low Frequency Power. (C) HF; High Frequency Power. (D) LF/HF ratio; Low Frequency Power to High Frequency Power ratio. Values are natural log transformed (except the LF/HF ratio) and are present as means ± SD.

All statistical procedures were carried out using IBM SPSS (Version 21 for Microsoft Windows, IBM Corporation, Armonk, New York). All data were checked for normality using the Shapiro–Wilks test; data were log transformed if normality was violated, this occurred for most HRV metrics and the plasma catecholamines (Figs. 1 and 2). Differences in participant demographics, baseline and exercising variables between groups were compared using independent *t*‐tests. The HRV metrics (baseline and paced breathing conditions) and plasma catecholamines (rest and exercise) were compared using mixed two‐way repeated measures ANOVA [factors: condition and group (T1D vs. CON)]. As no significant interactions (i.e., condition × group) were identified, Post Hoc analysis was not preformed, and the alpha values for condition, group, and interaction are presented in Figures 1 and 2. Separate Pearson's correlations and linear regressions were used to determine the association between the TP (determined at baseline) and the individual indicators of exercise capacity; *V̇*O_2max_, HR reserve and max HR in both T1D and CON. The HR reserve and maximum HR were model in addition to *V̇*O_2max_ as diabetes is known to impact the HR response to exercise. The LF/HF ratio was also considered with the same approach as TP. In all Pearson's and linear regression models the residuals were normally distributed with untransformed units, allowing for the use of these parametric models. In primary analysis three CON participants with exceedingly high TP (>11,000 msec^2^) were determined to be influential cases with Cook's distance values of between 1.2 and 79 (value > 1 being of concern). These participants were excluded from the presented secondary analysis which involved TP and the LF/HF ratio. In addition to the HRV metrics, other traditional (i.e., age, resting HR, and self‐reported exercise) and clinical variables (i.e., HbA_1c_) that would be available in clinical visits to exercise capacity were explored. Select linear regression analyses were interpreted (within the discussion) by multiplying the slope (*β*) of interest by the standard deviation of the regressed independent variable (i.e., TP), which calculates the associated change in the dependent variable (i.e., *V̇*O_2max_). An *P* < 0.05 was set to signify significance, and the data is presented as mean ± SD.

## Results

All 40 participants completed the study. Table 1 shows the participant characteristics, the T1D and CON were sex‐, age‐, mass‐, height‐ and self‐reported exercise‐ matched. Although T1D had a slightly greater BMI than CON (*P* = 0.06), both groups were within the same BMI range (i.e., classified overweight). Participants with T1D were diagnosed with T1D at a mean age of 16 year old (range: 1–42 year). The mean duration of T1D was 15 year (range: 1–46 year). All T1D were receiving insulin therapy (7 via insulin pump) and their mean HbA_1c_ was ~68 mmol/mol (~8.4%). Supplementary medical therapies included antidepressants (in 3 T1D and 1 CON), statins (in 4 T1D), ACE inhibitors (in 2 T1D), acetylsalicylic acid (in 1 T1D), omeprazole (in 1 T1D), antiepileptic (in 1 CON), and combined oral contraceptive (in 1 CON). All participants with T1D had no known significant microvascular complications; two participants had small changes in urine albumin. However, this may reflect variable glycemic control rather than actual diabetic nephropathy.

**Table 1:** Participant characteristics

Variable	T1D (*n *=* *23)	CON(*n *=* *17)	*P* value
Male to female ratio	12:11	9:8	0.96^a^
Age (year)	32 ± 13	32 ± 12	0.78
Mass (kg)	80.7 ± 14.7	75.9 ± 15.1	0.32
Height (cm)	170.2 ± 11.2	173.3 ± 7.3	0.30
BMI (kg/m)	27.9 ± 4.8	25.1 ± 3.9	0.06
Self‐reported exercise (h)	6.7 ± 9.8	5.7 ± 4.7	0.96
Age at T1D diagnosis (year)	16.4 ± 11.8	–	–
T1D duration (year)	15.2 ± 13.6	–	–
HbA_1C_ (mmol/mol)	68 ± 17	–	–

BMI, Body mass index; HbA_1c_, glycated hemoglobin.

Values are presented as mean ± SD.

a Chi‐squared test used for significance testing.

### Baseline parameters and CARTS

T1D had higher resting HR than CON (Table 2). However, resting BP, breathing frequency and catecholamines were similar between groups (Table 2 and Fig. 1). Three T1D participants had one abnormal CART result which was a depressed HR response to deep breathing. None of the remaining T1D or CON participants had any abnormal responses to the CARTs.

**Table 2:** Baseline and exercise variables

Variable	T1D (*n* = 23)	CON (*n* = 17)	*P* value
Resting HR (bpm)	74 ± 11	65 ± 9	<0.01
Max HR (bpm)	180 ± 13	186 ± 11	0.14
HR Reserve (bpm)	106 ± 16	122 ± 13	<0.01
Resting SBP (mm Hg)	123 ± 14	122 ± 10	0.77
Resting DBP (mm Hg)	75 ± 11	74 ± 11	0.75
Resting MBP (mm Hg)	91 ± 11	89 ± 9	0.73
Resting breathing frequency (br/min)	15 ± 5	13 ± 4	0.30
Max breathing frequency (br/min)	45 ± 7	54 ± 9	<0.01
Max tidal volume (L)	2.6 ± 0.7	2.8 ± 0.8	0.45
Max ventilation (L/min)	110 ± 32	132 ± 39	0.05
*V̇*O_2max_ (mL/min/kg)	32 ± 9	37 ± 9	0.07
Max R‐Value	1.29 ± 0.09	1.25 ± 0.08	0.12
Time to exhaustion (min)	8.7 ± 2.6	9.9 ± 2.0	0.14
Max workload (W)	186 ± 64	219 ± 57	0.06
Normalized max workload (W/kg)	2.3 ± 0.7	2.9 ± 0.7	0.01

Values are presented as means ± SD.

Resting HR, resting heart rate; max HR, maximum heart rate; HR reserve, heart rate reserve; SBP, systolic blood pressure; DBP, diastolic blood pressure; MBP, mean arterial blood pressure; *V̇*O_2max_, maximal aerobic capacity; max *R*‐value, maximum respiratory exchange ratio.

### HRV

The HRV metrics during baseline and paced breathing are shown in Figure 2. No significant interactions were found (Condition x Group), therefore Post Hoc testing was not performed. The T1D when compared to CON, had lower TP, LF power, and HF power, but not LF/HF ratio (Fig. 2). Regardless of group, most HRV metrics were lower during the paced than the baseline condition.

### Exercise capacity

All participants met the criteria for determination of *V̇*O_2max_. In T1D, *V̇*O_2max_ tended to be (~14%) lower than in CON (*P* = 0.07, Table 2). Some determinants of *V̇*O_2max_ were different between groups. In T1D, HR reserve was ~13% lower than CON as result of a significantly higher resting HR and a non‐significant reduction in max HR (Table 2). Additionally, in T1D max ventilation was ~17% lower, due a reduced max breathing frequency, as max tidal volume was not different between groups (Table 2). However the exercise levels of catecholamines and their change in response to exercise were not different between groups (Fig. 1).

### Associations with exercise capacity

**Figure 3:**
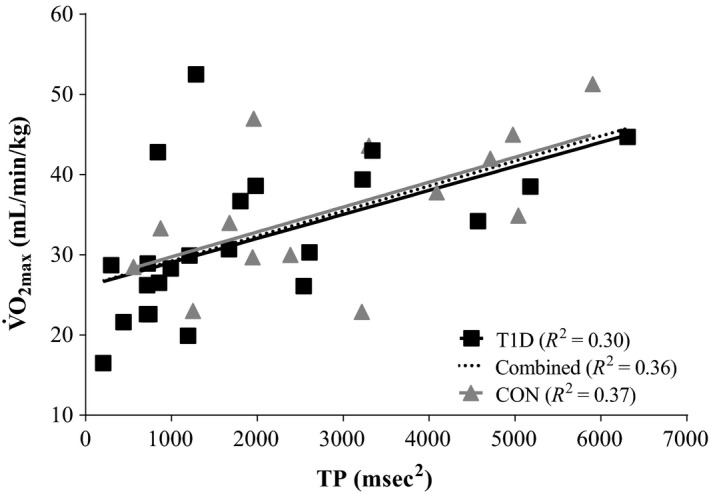
Association of Total Power (TP) to maximum aerobic capacity (*V̇*O_2max_). In controls (CON;* n* = 14) and in people with type 1 diabetes (T1D; *n* = 23). For *β*‐ and *P*‐values refer to Table 3. TP was derived from the baseline condition and is non‐transformed.

Separate correlation and regression analysis between exercise capacity (*V̇*O_2max_, HR reserve and max HR), HRV metrics (TP and the LF/HF ratio) and other traditional and clinical variables are shown in Table 3. Irrespective of group the relationship between TP and *V̇*O_2max_ was stronger than the other modeled independent variables. Specifically, TP was positively associated with *V̇*O_2max_ (T1D and CON), HR reserve (T1D and CON) and Max HR (T1D only). The *R* ^*2*^ between TP and *V̇*O_2max_ was 0.30 and 0.37 for T1D and CON, respectively (Table 3 and Fig. 3). By contrast the LF/HF ratio was not associated with *V̇*O_2max_, HR reserve or max HR in either group. Although T1D duration was associated with max HR (*R* ^2^ = 0.23), HbA_1c_ was not related to *V̇*O_2max_, HR reserve, or max HR. Irrespective of group, there were no associations between self‐reported exercise and exercise capacity.

**Table 3:** Associations with exercise capacity variables

Dependent variable	Independent variable	T1D		CON	
		R^2^	*β*	R^2^	*β*
*V̇*O_2_max (mL/min/kg)	TP (msec^2^)	**0.30** a	**0.003 ± 0.001** a	**0.37** a	**0.003 ± 0.001** a
	LF/HF ratio (au)	0.004	0.24 ± 0.85	0.002	0.20 ± 1.48
	Age (year)	**0.18** a	**−0.30 ± 0.14** a	**0.30** a	**−0.43 ± 0.17** a
	Resting HR (bpm)	0.12	**−**0.29 ± 0.17	0.15	**−**0.43 ± 0.26
	BMI (kg/m^2^)	**0.22** a	**−0.88 ± 0.36** a	0.12	**−**0.82 ± 0.58
	Self‐reported exercise (h)	0.048	0.4 ± 0.4	0.042	0.4 ± 0.5
	T1D duration (year)	0.02	**−**0.007 ± 0.012	–	–
	HbA_1c_ (mmol/mol)	0.0650	**−**0.14 ± 0.11	–	–
HR Reserve (bpm)	TP (msec^2^)	**0.41** a	**0.006 ± 0.002** a	**0.36** a	**0.004 ± 0.001** a
	LF/HF ratio (au)	0.009	**−**0.67 ± 1.50	0.002	**−**0.32 ± 1.84
	Age (year)	0.10	**−**0.38 ± 0.26	**0.42** a	**−0.69 ± 0.21** a
	Resting HR (bpm)	**0.35** a	**−0.88 ± 0.26** a	**0.24** a	**−0.73 ± 0.33** a
	BMI (kg/m^2^)	0.09	**−**0.99 ± 0.69	0.05	**−**0.71 ± 0.82
	Self‐reported exercise (h)	0.004	**−**0.2 ± 0.7	0.040	0.5 ± 0.7
	T1D duration (year)	0.05	**−**0.022 ± 0.021	–	–
	HbA_1c_ (mmol/mol)	0.0780	**−**0.27 ± 0.20	–	–
Max HR (bpm)	TP (msec^2^)	**0.40** a	**0.005 ± 0.001** a	0.08	0.002 ± 0.002
	LF/HF ratio (au)	0.002	0.24 ± 1.22	0.001	**−**0.17 ± 1.50
	Age (year)	**0.53** a	**−0.74 ± 0.15** a	**0.66** a	**−0.76 ± 0.14** a
	Resting HR (bpm)	0.01	0.12 ± 0.26	0.04	0.27 ± 0.33
	BMI (kg/m^2^)	0.08	**−**0.77 ± 0.57	0.04	**−**0.54 ± 0.73
	Self‐reported exercise (h)	0.040	**−**0.5 ± 0.6	0.013	0.3 ± 0.6
	T1D duration (year)	**0.23** a	**−0.038 ± 0.015** a	–	–
	HbA_1c_ (mmol/mol)	0.0001	**−**0.01 ± 0.17	–	–

Dependent variables: *V̇*O_2max_; maximum aerobic capacity; HR reserve, heart rate reserve; Max HR, maximum heart rate. Independent variables; TP, total power; LF/HF ratio, low frequency power to high frequency power ratio; resting HR, resting heart rate; BMI, body mass index; T1D; Type 1 diabetes, and HbA_1c_, glycated hemoglobin.

The *β* values are presented as the mean slope ± SD of the slope.

a Significant association (*P* < 0.05), which are in bolded text. TP and LF/HF ratio were derived from the baseline condition.

## Discussion

We examined whether resting cardiac autonomic modulation, characterized by HRV, was associated with exercise capacity in people with and without uncomplicated T1D. The main findings were as follows. First, despite a priori selection for uncomplicated T1D (i.e., free from known diabetes‐related chronic disease), subtle changes in resting cardiac autonomic modulation were evident in this population. These changes were not revealed using traditional diagnostic criteria (i.e., 2 or more abnormal CARTs) but were evident from resting HR (sub‐clinical change) and frequency‐domain analyses of resting HRV. Specifically, the T1D had higher resting HR and lower HRV (reductions in TP, LF and HF, but not LF/HF ratio) than matched CON. Second, the T1D tended to have a lower exercise capacity (HR reserve and near significant reduction in *V̇*O_2max_) than matched CON, as expected. Third, in accordance with our hypothesis TP was positively associated with exercise capacity in both groups. Indeed the attributed variance for TP was greater than other measured traditional indicators, such as age, resting HR, and self‐reported exercise.

### Effect of uncomplicated T1D on resting cardiovascular measures

In our uncomplicated T1D resting HR was elevated. This finding aligns with others (Bernardi et al. 2011; Spallone et al. 2011), who have shown elevation in resting HR across the T1D spectrum (i.e., uncomplicated to complicated). The ~11 beats/min elevation in resting HR observed in our uncomplicated T1D may have clinical implications, as a change of this magnitude is associated with an 18% increase in the risk of cardiovascular mortality in the general population (Ho et al. 2014). In contrast there was no difference in baseline BP between uncomplicated T1D and CON, which is frequently observed (Roy et al. 1989; Roberto et al. 2012), and both groups were within the normotensive range. Additionally, plasma catecholamines at rest, a global indicator of SNS status (Bernardi et al. 2011), were not different, which has been observed in those who have uncomplicated T1D (Nugent et al. 1997; Pop‐Busui et al. 2004).

Our interpretation of this resting data is that cardiac PNS modulation is reduced in uncomplicated T1D. An elevated resting HR is predominately caused by a reduced cardiac PNS modulation (Joyner 2016). Furthermore, three of our T1D participants had an abnormal heart response to deep breathing (one of the CARTs) which is attributed to impaired cardiac PNS modulation (Ewing and Clarke 1982; Vinik and Ziegler 2007; Vinik et al. 2013). When combined with the lack of between group differences in resting BP and plasma catecholamines, may indicate that SNS function was unlikely to be elevated in our uncomplicated T1D.

### Effect of uncomplicated T1D on resting HRV metrics

Our resting HRV findings also align with the notion that cardiac PNS modulation is reduced in uncomplicated T1D. Specifically, we found that TP, as well as LF and HF power were lower, while the LF/HF ratio was unchanged in uncomplicated T1D compared to CON. These changes are commonly observed across the spectrum of T1D (Colhoun et al. 2001; Vinik and Ziegler 2007). Such changes are used to highlight subtle early variation in cardiac autonomic modulation in patients with diabetes (Bernardi et al. 2011; Spallone et al. 2011), despite resting HRV being influenced by a number of factors including cardiorespiratory fitness. There appears to be a diabetes specific effect on resting HRV, as reductions in resting HRV metrics are apparent in newly diagnosed T1D without a decline in cardiorespiratory fitness (Röhling et al. 2017). Nevertheless, some of the controversy regarding frequency‐domain HRV analysis warrants consideration.

In the past, it has been argued that the LF band and the LF/HF ratio reflect cardiac SNS modulation and the balance of cardiac autonomic modulation, respectively (Eckberg 1997; Parati et al. 2006; Reyes del Paso et al. 2013; Joyner 2016). However, there is increasing recognition that the resting HRV metrics are more reflective of cardiac PNS than cardiac SNS modulation (Reyes del Paso et al. 2013; Joyner 2016). In line with this notion are the following three points: (1) high‐dose administration of the muscarinic receptor blocker, atropine (a PNS antagonist), abolishes most, if not all spectral power within all bands (TP, LF, and HF) (Eckberg 1997; Reyes del Paso et al. 2013; Joyner 2016); (2) SNS blockade does not influence the power within the LF band, whether it is presented in absolute or normalized units (Eckberg 1997; Reyes del Paso et al. 2013; Joyner 2016); (3) the power within the LF band does not correlate with myocardial noradrenaline spillover, which is a more direct quantifier of cardiac SNS modulation (Kingwell et al. 1994). In an attempt to circumvent these issues some authors argue that normalize of LF and HF power by dividing them by TP minus the power within the very‐low frequency band. The assumption is that normalization of LF and HF bands more accurately reflects cardiac SNS and cardiac PNS modulation respectively (Task Force, 1996). Others counter that normalization is inappropriate because normalized values are mathematically related to the LF/HF ratio, and therefore cannot be interpreted as a different physiological process (Eckberg 1997). Furthermore, normalization is also known to divorce LF and HF power from their suggested physiological relevance (Parati et al. 2006). Hence, normalization of LF and HF power were not carried out in this study. With the aforementioned implications of interpreting frequency‐domain resting HRV, we interpret any changes regardless of frequency‐band to reflect altered cardiac PNS modulation. Therefore the first order derivative TP from frequency‐domain analysis and not the second order derivatives (i.e., HF and LF) is used henceforth.

### Altered PNS in uncomplicated T1D

The idea of reduced cardiac PNS modulation in early and less complicated diabetes as indicated in this study with an elevation in resting HR and reduced resting HRV metrics as discussed above is not new. Classical work from Ewing et al. (1980), Ewing and Clarke (1982) indicated cardiovascular autonomic modulation changes induced by diabetes are progressive, with early damage to the PNS and then the addition of SNS damage in the later stages. The development and progression of cardiovascular autonomic dysfunction and/or neuropathy is poorly understood in T1D and often difficult to diagnose (Bernardi et al. 2011; Spallone et al. 2011). It is associated with diabetes duration, glycemic control, insulin resistance, and other factors (Karayannis et al. 2012; Vinik et al. 2013). The observed reduction in resting TP tended to be inversely associated with diabetes duration (*R* ^2^ = 0.14; *P* = 0.08), but not HbA_1c_ (*R* ^2^ < 0.01; *P* = 0.92), and the elevation in resting HR tended to be positively associated with HbA_1c_ (*R* ^2^ = 0.16; *P* = 0.06) but not diabetes duration (*R* ^2^ = 0.06; *P* = 0.27) in this study. Although speculative, cardiovascular autonomic dysfunction and neuropathy seem to mirror the development of peripheral neuropathy starting distally and progressing proximally (Pop‐Busui 2010). As the vagus nerve is the longest autonomic nerve and it is responsible for ~75% of all PNS activity, it stands to reason that early manifestation of cardiovascular dysfunction and/or neuropathy are associated with the PNS (Pop‐Busui 2010). Our participants with T1D had diabetes for at least a year, were receiving insulin therapy, and had an elevated HbA_1c_. Therefore it is conceivable that an effect on cardiac PNS modulation may have occurred in our uncomplicated T1D cohort.

### Effect of uncomplicated T1D on exercise capacity

We found that our uncomplicated T1D had blunted exercise capacity, which is common finding across the spectrum of T1D (Hilsted et al. 1979; Kahn et al. 1986; Roy et al. 1989; Hagglund et al. 2012; Peltonen et al. 2012; Koponen et al. 2013; Rissanen et al. 2015). Specifically in T1D, *V̇*O_2max_ tended (*P* = 0.07) to be reduced by ~5 mL/min/Kg, which equates to ~1.4 metabolic equivalents (METs). The Fick equation states that a change in *V̇*O_2max_ could arise from altered cardiac response, and/or peripheral extraction (arterial – venous O_2_ difference). Peripheral oxygen extraction was not assessed in this study, however it is unlikely to contribute to the observed reduction in *V̇*O_2max_, as peripheral oxygen extraction seems to be unaffected by T1D (Rissanen et al. 2015), despite lower oxidative capacity in muscles (Crowther et al. 2003). This then highlights the importance of the cardiac response contributing to *V̇*O_2max_ and exercise tolerance. We found that HR reserve (i.e., reduced cardiac response) was ~13% lower in T1D. While HR reserve is not often quantified in T1D, the combination of elevated resting and lower max HR's would cause the reduced HR reserve. We found that max HR in T1D was ~6 bpm lower though not significantly so (*P* = 0.14). Max HR generally seems to be unchanged with less complicated T1D (Baldi et al. 2010; Hagglund et al. 2012; Roberto et al. 2012; Koponen et al. 2013; Rissanen et al. 2015). The exercise level of plasma catecholamines and their responses exercise were not affect by T1D (Fig. 1), this has been previously observed (Nugent et al. 1997; Pop‐Busui et al. 2004), and may indicate a similar global SNS response to exercise in uncomplicated T1D.

The cause(s) of a blunted exercise capacity in T1D are numerous and complex, a complete discussion or elucidation of these with further physiological testing are outside the scope of this study. However, exercise capacity in T1D is known to be affected by glycemic control (Baldi et al. 2010; Rissanen et al. 2015), cardiovascular autonomic dysfunction and/or neuropathy (Hilsted et al. 1979; Kahn et al. 1986; Roy et al. 1989), insulin resistance (Nadeau et al. 2010), and there are likely to be other factors. The level of HbA_1c_ (range: 49–111 mmol/mol) was not associated with *V̇*O_2max_, HR reserve, or max HR in uncomplicated T1D (Table 3), as previously reported (Koponen et al. 2013). However, highly‐trained T1D athletes with poorer glycemic control (mean HBA_1c_: ~62 mmol/mol vs. ~48 mmol/mol) had blunted *V̇*O_2peak_ and peak HR (Baldi et al. 2010). It has since been suggested that appropriate glycemic control may independently increases exercise capacity in people with T1D (Baldi and Hofman 2010), which is not supported in this study. It is difficult to know why this study in non‐athletes with uncomplicated contradicts these findings, and warrants further investigation.

### Associations of resting HRV with exercise capacity

A focus of this study was to use the surrogate indicator of resting cardiac PNS modulation; TP and its separate associations to *V̇*O_2max_, HR reserve and max HR in those with and without uncomplicated T1D to indicate exercise capacity. The associations between TP and the exercise capacity variables were stronger than traditional indicators such as age, resting HR, and self‐reported exercise (Table 3). Specifically TP, which was reduced in our uncomplicated T1D, was associated with *V̇*O_2max_ (T1D: *R* ^2^ = 0.30; & CON: *R* ^2^ = 0.37; Fig. 3) and HR reserve (T1D: *R* ^2^ = 0.41; & CON: *R* ^2^ = 0.36) in both T1D and CON, and max HR in T1D only (*R* ^2^ = 0.40; Table 3). A one standard deviation increase in TP (T1D: 1646 msec^2^; & CON: 1724 msec^2^) when multiplied by the slope (*β*) of the relationship was associated with an increase in *V̇*O_2max_ of 4.9 mL/min/Kg in T1D and 5.2 mL/min/Kg in CON. Resting TP, regardless of group was associated with *V̇*O_2max_ and HR reserve, indicating a continuum, where the participants with uncomplicated T1D are clustered with lower values of TP than CON (as highlighted in Fig. 3).

Despite associations between HRV metrics and exercise capacity in other populations (for review see (Hautala et al. 2009)), these are not well documented in uncomplicated T1D. Additionally, no studies have compared the strength of the associations between resting HRV metrics and exercise capacity to those between traditional and clinical indicators and exercise capacity. Our findings contradict Hagglund et al. (2012) who did not find an association in resting HRV and *V̇*O_2max_ in their T1D group, despite both studies examining those whom seem to have uncomplicated T1D. However, there are two major differences between the studies. First, differences in statistical power (smaller sample in (Hagglund et al. 2012)); and second, the use of HF power (a second derivative frequency‐domain metric) compared to TP in our study. In support of our findings a recently published study has reported associations between HRV metrics and exercise capacity in newly diagnosed T1D (Röhling et al. 2017).

Although speculative, an explanation of why a higher resting TP is associated with exercise capacity might be that it is reflective of a “healthier” cardiac autonomic system (Thayer and Lane 2007; Joyner and Green 2009). Commonly it is suggested that a strong and efficient PNS, but not an overactive SNS, is the basis of a well‐balanced and adaptable cardiac autonomic system (Thayer and Lane 2007; Joyner and Green 2009). During exercise it is known that a shift in the balance of cardiac autonomic modulation (PNS:SNS ratio) from a ratio of ~4:1 at rest to ~1:4 at maximal exercise is required to increase HR (White and Raven 2014). Furthermore, relationships between aerobic exercise training and/or higher aerobic capacity and enhanced cardiac PNS modulation are known in healthy populations (Billman 2002; Buch et al. 2002; Carter et al. 2003; Hautala et al. 2009), and conversely, those who suffer from autonomic neuropathy have blunted exercise capacity (Hilsted et al. 1979; Kahn et al. 1986; Roy et al. 1989; Vinik and Ziegler 2007). Therefore it appears that a well‐balanced resting cardiac autonomic nervous system with predominant PNS modulation is an important factor in influencing exercise capacity not only in CON but in uncomplicated T1D alike.

### Clinical implications

Our study suggests that people with uncomplicated T1D can show subtle changes in autonomic nervous system function, which seem to be reflected in increased resting HR and reductions in frequency‐domain HRV metrics, the latter which can be an additional test within a standard clinical visit. These subtle changes were not conclusively detected by the traditional CARTs, possibly indicating these tests may not be sensitive or specific enough to detect these alterations. The associations between TP and measures of exercise capacity in both T1D and CON were generally stronger that the other traditional and clinical indicators. Specifically a one standard deviation change in TP was associated with an increase in *V̇*O_2max_ of 4.9 mL/min/Kg in T1D and 5.2 mL/min/Kg in CON. This indicates that TP, in people with and without uncomplicated T1D may provide useful information in regard to their capacity to exercise.

In summary, resting cardiac PNS modulation and exercise capacity were reduced with uncomplicated T1D. TP was positively associated with *V̇*O_2max_ (*R* ^2^ ≥ 0.3) in people with and without uncomplicated T1D and these relationships were stronger than other more commonly used traditional indicators, albeit within a modest cohort. This study highlights the importance of resting PNS modulation of the cardiovascular system in the adaptation to stressful stimuli like exercise.

## Conflict of Interest

The author(s) have no conflict of interest to declare.
